# Selected Articles

**Published:** 1866-08

**Authors:** 


### SELECTED ARTICLES.

We have noticed of late a disposition in certain medical gentlemen to court notoriety in a way that strikes us as not only very unprofessional but in very bad taste. We now not unfrequently see a notice of a "very difficult and dangerous operation," performed by a very skilful Surgeon A; of a very learned and scientific lecture on some rare (?) form of disease, by Dr. B; of some astonishing recovery brought about by the assiduous attentions of the eminent Dr. C; and we cannot make ourselves believe that such reports can find their way to the public without the knowledge of the principals. The extent to which this system is now being carried on is shameful, and deserves the serious attention of some Committee on Medical Ethics. It may, perhaps, be that the public are particularly anxious to know of the exploits of Dr. A., the views of Dr. B., and the success of Dr. C.; but it does seem strange that some of the doings of *our really substantial* men are not alike so faithfully and regularly reported.

The anxiety of the public to know something of cholera has tempted many an otherwise prudent man into the indiscretion of addressing a letter on the subject to some of our leading papers. There would be some shadow of an excuse for this if these gentlemen could be considered authorities in the matter; but, unfortunately for us, their ideas are crude in the extreme, and while they may tend to glorify the writer, inevitably stultify the profession. If the public desire to have any authoritative opinions concerning this disease, they can appeal to the Health Board and be satisfied; it is the duty of this body to minister to the wants of the community in this respect; and, so far as we can see, they do their duty.

Medical men occupying public offices have a right to address the public, to whom they are expected to give an account of themselves; there may be instances too, where late incumbents may, from necessity, rush into print to defend views before mooted in public; but it strikes us as discreet, at least, for other members of the profession to wait until their opinion is sought for, and not to cheapen their otherwise valuable services by volunteering too much.—*N. Y. Med. Record*, July 16, 1866.

---

*A Splinter Embedded for Seven Years in the Muscles of the Forearm Without Causing Suppuration.*

Editor Medical and Surgical Reporter,—As the following case is an unusual one, and as its report will occupy but little of your space, I place it at your disposal:

Mr. G., of Kenosha county, called to consult me a few days ago, in relation to an enlargement, existing upon the anterior aspect of the right forearm. Seven years ago, in jumping from a hayrack, he struck the arm upon the splintered end of an oak stake, projecting from the side of the rack, inflicting a lacerated wound, an inch or more in length. It healed kindly in a few weeks, but there remained an enlargement, reaching from the beginning of the lower third of the arm, to the carpal articulation, well filling the space between the ulna and radius. Firm pressure at each end, and in its course, gave the impression that a foreign body of some kind had become safely lodged there, and the history given, suggested the possibility of its being a piece of the oak stake before mentioned. But was it possible, for a piece of *wood*, to remain embedded in the flesh for a period of *seven years*, and at no time to produce suppuration? This seemed impossible, but true it was, for a free incision through its extent, enabled me to remove a splinter of oak, two inches in length, and in circumference equal to a female catheter. It was a little darkened in color and polished like glass.

Racine, Wis., June 30th, 1866. J. G. MEACHEM, M. D.

